# Complete genome sequence of *Bacteroidales* bacterium strain MB20-C3-3 isolated from sewage sludge

**DOI:** 10.1128/mra.01396-25

**Published:** 2026-01-29

**Authors:** Wanling Qiu, Guowen Dong, Jingjing Zhao, Yin Li, Chao-Jen Shih, Yen-Chi Wu, Chih-Hung Wu, Shu-Jung Lai, Wangchung Xiao, Wei-Ling Zhang, Lintao Wu, Song Wang, Hangying Zhang, Sheng-Chung Chen

**Affiliations:** 1 College of Environment and Safety Engineering, Fuzhou University 562589, Fuzhou, Fujian, People’s Republic of China; 2 School of Resources and Chemical Engineering, Sanming University 659925, Sanming, Fujian, People’s Republic of China; 3 Fujian Provincial Key Laboratory of Resources and Environmental Monitoring and Sustainable Management and Utilization, Sanming University 66283, Sanming, Fujian, People’s Republic of China; 4 College of Resources and Environment, Fujian Agriculture and Forestry University 602381, Fuzhou, Fujian, People’s Republic of China; 5 School of Chemistry and Materials, Fujian Normal University 12425, Fuzhou, Fujian, People’s Republic of China; 6 Bioresource Collection and Research Center, Food Industry Research and Development Institute 63418, Hsinchu, Taiwan, Republic of China; 7 Graduate Institute of Biomedical Sciences, China Medical University 599553 , Taichung City, Taiwan, Republic of China; 8 Research Center for Cancer Biology, China Medical University 38019, Taichung City, Taiwan, Republic of China; Nanchang University, Nanchang, Jiangxi, China

**Keywords:** anaerobes, sewage sludge, *Bacteroidales*

## Abstract

Here, we report the complete genome sequence of *Bacteroidales* bacterium strain MB20-C3-3 (=BCRC 81427) isolated from sewage sludge. The genome is 2,379,874 bp in length with a guanine and cytosine content of 40.0%. The genome of strain MB20-C3-3 was used for further species delineation and comparative genomic analyses.

## ANNOUNCEMENT

**Fig 1:**
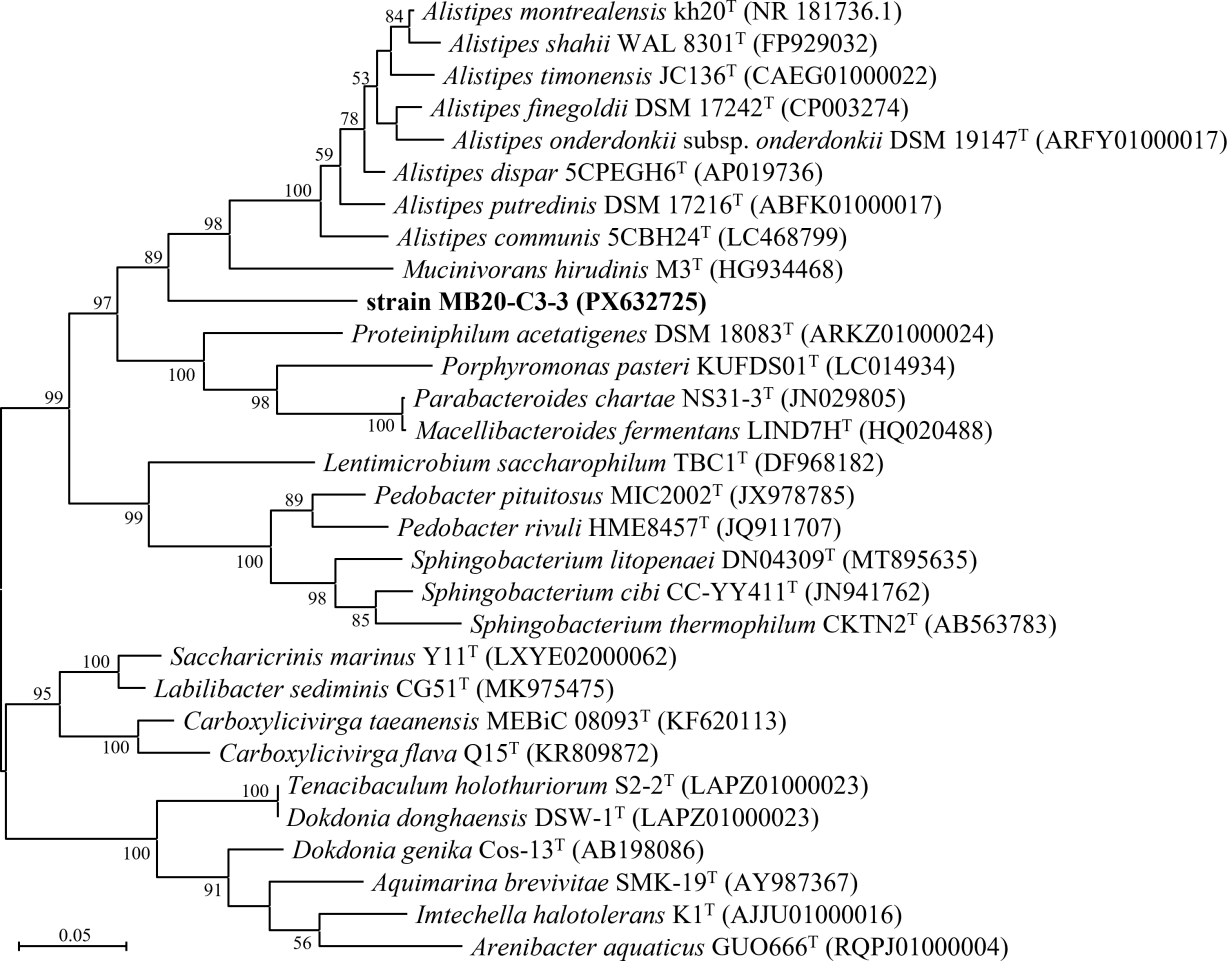
Maximum likelihood tree based on 16S rRNA gene sequences of strain MB20-C3-3 and related taxa. Sequence alignment was performed using Clustal W (6), and the Tamura-Nei model (7) was applied as the nucleotide substitution model. Bar, 0.01 substitutions per nucleotide position. Bootstrap values were expressed as percentages of 1000 replicates.

Strain MB20-C3-3 was isolated from the sewage sludge of the Wastewater Treatment Plant of Sanming Steel Co., Ltd., Fujian, China. Sewage sludge was collected on 25 June 2021. The sample of sludge was inoculated into the anaerobic modified DSM 924 medium (per liter: 1 g MgCl_2_·7H_2_O, 0.5 g KCl, 0.1 g CaCl_2_·2H_2_O, 0.4 g K_2_HPO_4_, 1 g NH_4_Cl, 10 mL trace element solution, 2 g yeast extract, 2 g tryptone, 0.5 mL Na-resazurin solution [0.1%], 4 g NaHCO_3_, 10 mL vitamin solution, 0.25 g L-cysteine-HCl·H_2_O, 0.25 g Na_2_S·9H_2_O, and headspace: N_2_:CO_2_ = 4:1) prepared according to the instruction for the medium and incubated at room temperature (~25°C) for 2 weeks. Strain MB20-C3-3 was purified by serial dilution and the rolling-tube technique (1) using the modified DSM 924 medium at 25°C. The isolate was identified by 16S rRNA gene clone sequencing with 8F (5′-AGAGTTTGATCCTGGCTCAG-3′) and 1492RU (5′-TTTTAATTAAGGTTACCTTGTTACGACTT-3′) primers (2). The purity of bacteria was confirmed by morphological observation, 16S rRNA gene analysis, and genome sequencing. Based on BLASTN analysis (3), strain MB20-C3-3 (16S rRNA gene: PX632725) showed the highest similarity (86.82%) to *Alistipes montrealensis* kh20^T^ (4). Phylogenetic analysis of 16S rRNA gene sequences performed by MEGA11 (5) for strain MB20-C3-3 and related taxa indicated that strain MB20-C3-3 could be affiliated with a novel genus (Fig. 1). The genome of strain MB20-C3-3 was selected for sequencing for species delineation and comparative genomic analyses.

The strain MB20-C3-3 has been deposited in the Bioresource Collection and Research Center, Taiwan as strain BCRC 81427. It was grown in the modified DSM 924 medium and incubated at 30°C. The genomic DNA of strain MB20-C3-3 was extracted using a modified method of Johnson (8) and Jarrell et al. (9). Briefly, cells from a 500 mL culture were lysed with 1% SDS, and DNA was purified by phenol-chloroform extraction and ethanol precipitation, then quantified by UV-vis spectrophotometry. The extraction yielded approximately 30 µg of high-quality genomic DNA.

Whole-genome sequencing was performed using Illumina Novaseq 6000 platform and Oxford Nanopore MinION (Guangdong Magigene). For Illumina sequencing, approximately 100 ng of DNA was used for library preparation following the requirements of the ALFA-SEQ DNA Library Prep Kit (FINDROP, Guangzhou), assessed by Qubit 4.0 fluorometer (Life Technologies) and Qsep400 (Houze Biological Technology), and sequenced to generate 150 bp paired-end reads. Raw reads were processed with fastp v0.23.4 (10) to remove adapters, trim low-quality bases, and discard short fragments, yielding 9,515,760 clean reads (Table 1).

**TABLE 1:** Genome characteristics of *Bacteroidales* bacterium strain MB20-C3-3

Characteristic	Value
Sequencing and assembly features	
No. of Illumina clean reads	9,515,760
No. of Nanopore clean reads	239,640
Total no. of Illumina bases	1,423,176,381 bp
Total no. of Nanopore bases	1,247,571,425 bp
Genome coverage (Illumina; Nanopore)	598×; 524×
Genome features	
Chromosome size (GC content)	2,379,874 bp (40.0%)
Total no. of genes	2,158
No. of coding sequences	2,108
No. of pseudogenes	14
No. of rRNAs	9
No. of tRNAs	38
No. of noncoding RNAs	3

For MinION sequencing, a ligation-based library (SQK-LSK109) was prepared from 1 µg of unsheared genomic DNA (>1 kb) and sequenced on an R9.4.1 flow cell. MinION reads were base-called using Guppy v6.5.7 in high-accuracy mode, which also performed built-in adapter trimming. Read quality control and filtering were conducted with NanoFilt v2.8.0 (11) to remove low-quality and short reads, yielding 239,640 reads (*N*_50_ = 13,202 bp; average = 5,206 bp; total = 1,247,571,425 bp; Table 1).

Hybrid *de novo* assembly was performed with Unicycler v0.5.0 (12), which identified overlaps, trimmed contig ends, and confirmed circular topology without rotating the genome. The assembly generated a circular genome of 2,379,874 bp with 40.0% guanine and cytosine (GC) content (Table 1). The average short- and long-read coverages were 598× and 524×, respectively. The genome assembly was annotated using PGAP v6.6 (13) upon deposition to the National Center for Biotechnology Information. Default parameters were used for all software tools.

This genome represents a deeply branching *Bacteroidales* lineage. Its distinctive features, including oxidoreductases (WRQ33061, WRQ33102), stress-response genes (WRQ34105, WRQ33080), and ion transporters (WRQ34102, WRQ33053), expand the phylogenetic and functional diversities of this understudied order.

## Data Availability

The genome sequence of strain MB20-C3-3 has been deposited in GenBank under accession number CP141202. The version of the genome described in this paper is the first version. The BioProject and BioSample accession numbers are PRJNA1053856 and SAMN38876749. Illumina Novaseq and MinION raw reads were deposited in the Sequence Read Archive (SRA) under accession numbers SRR36230458 and SRR36230459, respectively. The accession number of the 16S rRNA gene is PX632725.
